# Pertussis Maternal Immunization: Narrowing the Knowledge Gaps on the Duration of Transferred Protective Immunity and on Vaccination Frequency

**DOI:** 10.3389/fimmu.2017.01099

**Published:** 2017-09-06

**Authors:** María Emilia Gaillard, Daniela Bottero, María Eugenia Zurita, Francisco Carriquiriborde, Pablo Martin Aispuro, Erika Bartel, David Sabater-Martínez, María Sol Bravo, Celina Castuma, Daniela Flavia Hozbor

**Affiliations:** ^1^Laboratorio VacSal, Instituto de Biotecnología y Biología Molecular (IBBM), Facultad de Ciencias Exactas, Universidad Nacional de La Plata, CCT-CONICET La Plata, La Plata, Argentina

**Keywords:** pertussis, *Bordetella pertussis*, pregnancy immunization, acellular vaccine, protection

## Abstract

Maternal safety through pertussis vaccination and subsequent maternal–fetal-antibody transfer are well documented, but information on infant protection from pertussis by such antibodies and by subsequent vaccinations is scarce. Since mice are used extensively for maternal-vaccination studies, we adopted that model to narrow those gaps in our understanding of maternal pertussis immunization. Accordingly, we vaccinated female mice with commercial acellular pertussis (aP) vaccine and measured offspring protection against *Bordetella pertussis* challenge and specific-antibody levels with or without revaccination. Maternal immunization protected the offspring against pertussis, with that immune protection transferred to the offspring lasting for several weeks, as evidenced by a reduction (4–5 logs, *p* < 0.001) in the colony-forming-units recovered from the lungs of 16-week-old offspring. Moreover, maternal-vaccination-acquired immunity from the first pregnancy still conferred protection to offspring up to the fourth pregnancy. Under the conditions of our experimental protocol, protection to offspring from the aP-induced immunity is transferred both transplacentally and through breastfeeding. Adoptive-transfer experiments demonstrated that transferred antibodies were more responsible for the protection detected in offspring than transferred whole spleen cells. In contrast to reported findings, the protection transferred was not lost after the vaccination of infant mice with the same or other vaccine preparations, and conversely, the immunity transferred from mothers did not interfere with the protection conferred by infant vaccination with the same or different vaccines. These results indicated that aP-vaccine immunization of pregnant female mice conferred protective immunity that is transferred both transplacentally and *via* offspring breastfeeding without compromising the protection boostered by subsequent infant vaccination. These results—though admittedly not necessarily immediately extrapolatable to humans—nevertheless enabled us to test hypotheses under controlled conditions through detailed sampling and data collection. These findings will hopefully refine hypotheses that can then be validated in subsequent human studies.

## Introduction

Pertussis or whooping cough is a respiratory disease mainly caused by the Gram-negative coccobacillus *Bordetella pertussis*. This disease affects all individuals regardless of age, but with higher morbidity and mortality rates among infants that have received either no vaccine or an incomplete vaccination schedule (1–3). Pertussis has resurged as a major public health concern in many countries (4, 5). Until two decades ago, the control of the disease was mainly carried out through a vaccination scheme consisting in a three-dose primary series, with the first dose administered as early as at 6 weeks of life, with subsequent doses being completed by 6 months of age (6). In order to accomplish the three-dose primary series, two types of vaccines are currently available: a whole-cell vaccine based on standardized cultures of *B. pertussis* strains (wP) and an acellular form [acellular pertussis (aP)] composed of purified *B. pertussis* immunogens. Acellular vaccines, originally developed to reduce the side effects associated with wP vaccination (7, 8), have since replaced wP, especially in industrialized countries. Unfortunately, the duration of the immunity conferred by these two vaccines is not lifelong (9). Moreover, recent data indicated that protection from aP vaccines wears off faster than that induced by wPs. This weakness in the current vaccines together with the lack of optimal vaccination coverage and the evolution of the causal agent to greater vaccination resistance have contributed to the recent rise in *pertussis* incidence and fatalities (10–12). While coverage is improved and better vaccines are designed, many countries have added vaccination boosters beyond the primary doses with the main aim at reducing both the disease burden and the incidence in the most vulnerable populations.

Maternal pertussis immunization during the third trimester of every pregnancy is one of the recent strategies recommended in several countries to improve pertussis control in infants (13, 14). The reported safety of the acellular vaccine when used during pregnancy and the placental transfer of *pertussis* antibodies from mothers to their infants that has been detected argue in favor of this strategy (15–17). Nevertheless, the question of whether or not this approach is able to effectively protect neonates against pertussis and how the transmitted maternal immunity affects the protection conferred by subsequent infant vaccination are still insufficiently clear. Recently, Amirthalingam et al. reported the effectiveness of maternal immunization in preventing infant pertussis, as evaluated 1 year after the introduction of the maternal-*pertussis*-immunization program in England in 2012 (18). Moreover, in the 3 years following its introduction vaccine effectiveness against confirmed pertussis has been sustained >90% with a vaccine effectiveness against infant deaths estimated at 95% (95% confidence interval, 79–100%). Furthermore, the authors reported that the protection conferred by maternal immunization was retained in infants who received the first dose of the primary series (18). Though all these data are highly promising and support the successfulness of maternal immunization, the reported number of cases in that study was, unfortunately, small; and therefore an ongoing assessment is still needed.

The use of animal models has also indicated an effective protection against pertussis in the offspring of mothers immunized during pregnancy. In a primate model (baboons), it was observed that both maternal and neonatal vaccinations were found to confer protection against the pathogen (19). The authors of those baboon experiments suggested that the transmitted maternal antibodies alone would be sufficient to confer protection against pertussis symptoms. In pigs, protection seems to be related to the presence of anti-*B. pertussis* antibodies in the colostrum (20, 21). We need to emphasize here that although in mice a protection transmitted to the pups *via* the placenta has been duly demonstrated, a transmission *via* the breast milk cannot be discarded since that *via* has not yet been completely investigated. Oda et al. (22) and Quinello et al. (23) reported some data on that topic.

We thus used this mouse model in order to substantially enhance our understanding of the efficacy of maternal pertussis immunization in the protection of subsequent offspring as well as determine the potential interference of maternal immunity with the eventual protection of those offspring by the primary vaccination against *B. pertussis*.

## Materials and Methods

### Mice

BALB/c mice (4 weeks old), obtained from the Instituto Biológico Argentino SAIC (Biol Argentina), were kept in ventilated cages and housed under standardized conditions with regulated daylight, humidity, and temperature. The animals received food and water *ad libitum*. Day 1 of gestation was determined when vaginal plugs were observed. Breeding cages were checked daily for new births, and the pups were kept with their mothers until weaning at the age of 4 weeks. The animal experiments were authorized by the Ethical Committee for Animal Experiments of the Faculty of Science at La Plata National University (approval number 004-06-15 and 003-06-15).

### *B. pertussis* Strain and Growth Conditions

*Bordetella pertussis* Tohama phase I strain CIP 8132 was used throughout this study as the strain for challenge in the murine model of protection. *B. pertussis* was grown in Bordet–Gengou agar supplemented with 15% (v/v) defibrinated sheep blood (BG-blood agar) for 72 h at 36.5°C. Isolated colonies were replated in the same medium for 24 h and then resuspended in phosphate-buffered saline (PBS: 123 mM NaCl, 22.2 mM Na_2_HPO_4_, 5.6 mM KH_2_PO_4_ in MilliQ^®^ nanopure water; pH 7.4). The optical density (OD) at 650 nm was measured and serial 10-fold dilutions plated onto BG-blood agar to determine the density of the challenge inoculum.

### Vaccines

The maternal immunization protocols were performed with the three-valent pertussis aP BOOSTRIX^®^ (GSK, GlaxoSmithKline), with composition per human dose (HD): pertussis toxoid (8 µg), pertactin (2.5 µg), filamentous hemagglutinin (8 µg), tetanic toxoid (20 IU), and diphtheria toxoid (2 IU). For all experiments, immunization was carried out through the use of a 1/10 HD of that vaccine, hereafter referred to as a mouse dose (MD). The vaccinations of infant mice were performed with 1 MD of the aP, a commercial wP vaccine (DTP vaccine, PT. BIO FARMA, Indonesia), or the *B. pertussis*-outer-membrane-vesicle-based vaccine formulated by us as previously described (24), to be referred to as the OMV vaccine.

### Experimental Protocol

#### Maternal Immunization and Offspring Protection

Female BALB/c mice (*n* = 10) were intraperitoneally immunized with three doses of commercial acellular vaccine (aP) Boostrix™ 1/10 HD at days 0 and 14. Before applying the third vaccine, dose females were housed with males within the same cage and daily checked for pregnancy, when mucosal vaginal plug was detected a third vaccine dose was applied. Pregnancy eventually occurs after detection of vaginal mucosal plug. Mice couples stayed cohoused until the end of the experiment. Non-immunized mice were used as negative control of protection. Offspring born to either immunized or non-immunized mothers were intranasally challenged with a sublethal dose (10^6^–10^8^ CFU 40 µl^−1^) of *B. pertussis* Tohama phase I at 21 days of life. Seven days after challenge, mice were sacrificed, and their lungs were harvested, homogenized in PBS and plated in serial dilutions onto BG-blood agar to count CFUs after incubation at 37°C for 3–4 days. At least three independent assays were performed.

#### Passive Immunization through Lactation

To investigate the protection of infant mice by means of passive immune transfer through lactation; after giving birth, aP-vaccinated mothers were separated from their own pups and exchanged with non-immunized mothers that had given birth at the same time. The changeling pups were then breast-fed by the surrogate mothers until weaning at the age of 4 weeks. Finally, the mice were infected with *B. pertussis* and protection assessed on day 7 as described above.

#### Adoptive Transfer

To study protection conferred by passive transfer, pooled serum (100 µl) or spleen cells (20–50 × 10^6^) from mice born from non-immunized or immunized dams were transferred intraperitoneally to 4-week-old naïve mice. Twenty-four hours later, the mice were infected with *B. pertussis* and protection assessed on day 7 as described above.

#### Effect of Infant Vaccination on Protection in Mice Born to Vaccinated Mothers

To study the effect of active immunization of infant mice born to vaccinated mothers on protection from subsequent pertussis infection, the offspring were immunized at 4 weeks of age with an MD of the commercial aP, a commercial wP vaccine, or with the OMV vaccine. Non-immunized offspring from aP-immunized mothers or aP-immunized mice at 4 weeks of age were used as controls. Mice were challenged with *B. pertussis* 2 weeks after receiving the vaccine dose and protection assessed on day 7 as described above.

### Enzyme-Linked Immunosorbent Assay

As we previously described (25), plates (Nunc A/S, Roskilde, Denmark) were coated with sonicated *B. pertussis* Tohama phase I (whole-cell lysates), designated Bp, or with the purified recombinant pertussis toxin (PTxA), each at 3 µg/ml in 0.5 M carbonate buffer pH 9.5, by means of an overnight incubation at 4°C. The rinsed plates were then blocked with 3% milk in PBS (2 h 37°C) and incubated with serially diluted samples of mouse serum (1 h 37°C). In the experiments described above, the samples of blood used were collected from mothers at delivery, from mothers and pups at weaning, from mothers and pups before pup challenge, and from pups 13 days after immunization. The sera were obtained after leaving the blood samples to clot for 1 h at 37°C followed by centrifuging for 10 min at 7,000 × *g*. IgGs from individual serum or pooled sera bound to the plates were detected after a 2-h incubation with goat anti-mouse-IgG-linked horseradish peroxidase (1:8,000 Invitrogen, USA). As substrate 1.0 mg/ml *o*-phenylenediamine (Bio Basic Canada, Inc.) in 0.1 M citrate-phosphate buffer, pH 5.0 containing 0.1% hydrogen peroxide was used. ODs were measured with Titertek Multiskan Model 340 microplate reader (ICN, USA) at 492 nm, and the OD was plotted as a function of the log of the (serum dilution)^−1^. A successful assay produced for each antibody sample a sigmoidal curve in this type of plot. The titer of each antibody sample was determined from this curve by identifying by GraphPad Prism^®^ software the concentration (expressed as inverse of the dilution of the antibody) that provokes a half way between the basal response and the maximal response.

Of the experimental protocol—it performed in triplicate—one representative experiment is presented in Section “Results.”

### Statistical Analysis

The data were evaluated statistically by one-way analysis of variance followed by the Tukey test *post hoc* (*via* the GraphPad Prism^®^ software). Differences were considered significant at a *p* < 0.05.

## Results

### Maternal Immunization and Protection of the Offspring against *B. pertussis* Infection

**Figure 1:**
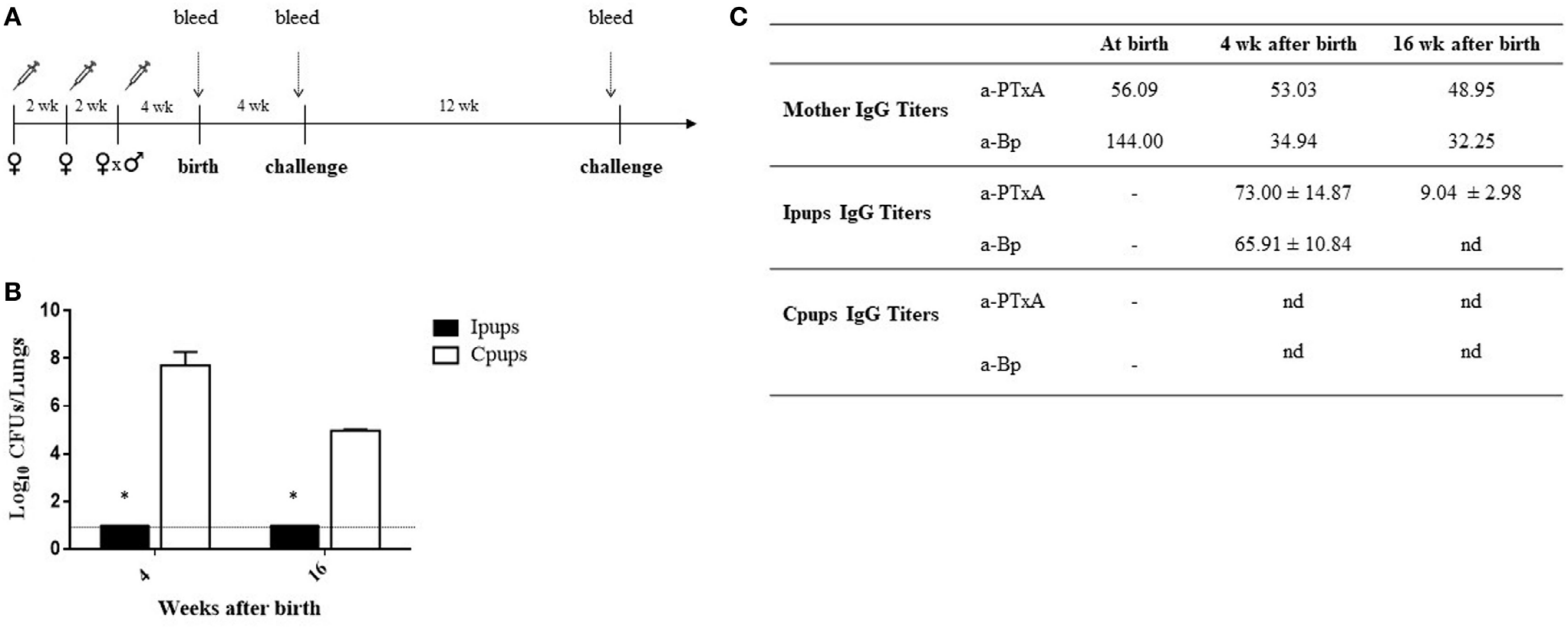
Effect of maternal immunization on protection of offspring against *Bordetella pertussis* infection. **(A)** Schematic representation of the maternal vaccination and challenge protocols. Female mice were vaccinated twice with a commercial acellular pertussis (aP) at a 2-week interval; mated with male mice; and when the vaginal plug was detected, a third aP dose was administered. Non-immunized females were mated with male mice at the same time as the immunized females. Mice were born 3–4 weeks after the last immunization. Mice born to aP-immunized or non-immunized females (controls) were challenged with *B. pertussis* at 4 and 16 weeks after birth. Mothers and pups were bled at different time points as indicated by the dotted vertical arrows. **(B)** Short- and long-term protection of offspring through maternal immunization. Mice born to aP-immunized (Ipups *n* = 6, black bars) or non-mmunized (Cpups *n* = 6, white bars) females were challenged with *B. pertussis* Tohama I at 4 and 16 weeks after birth. The number of bacteria recovered from mouse lungs, expressed as the log_10_ of CFUs per lungs, is plotted on the *ordinate* for the times after birth in weeks indicated on the *abscissa*, with the data representing the means ± the SD. The dotted horizontal line indicates the lower limit of detection. *p < 0.001 Ipups versus Cpups. **(C)** The anti-whole-cell-*B. pertussis* (a-Bp)- and anti-pertussis toxin (a-PTxA)-specific IgG titers were determined in the mother and in the offspring at the indicated time points. The titers are expressed as the geometric mean of the data from each group. *p* < 0.001 a-PTxA and a-Bp IgG titers in Ipups at 4 weeks after birth versus those detected at 16 weeks after birth. The results from one representative experiment are shown. nd, not detected.

To evaluate the protection of the offspring against *B. pertussis* infection through a maternal-vaccine-induced immunization, female mice were vaccinated twice with a commercial aP within an interval of 2 weeks, then mated with male mice and a third aP-vaccine dose administered when a vaginal plug was detected. Non-immunized females were mated with male mice at the same time to serve as the negative-control dams. Mice born to aP-immunized (hereafter referred to as Ipups) or non-immunized females (hereafter referred to as Cpups, for negative control) were challenged (intranasally) with 10^6^ CFUs of *B. pertussis* at 4 and 16 weeks after birth (Figure 1A). We observed significant differences in the lung *B. pertussis* bacterial counts between mice born to immunized mothers and the negative-control group (Figure 1B; *p* < 0.001). Differences in CFUs of six to seven orders of magnitude were detected between the Ipups and the Cpups challenged at 4 weeks after birth and of about four to five orders of magnitude after challenge at 16 weeks of age. Once again, the CFU counts in the Ipups were non-detectable. What is notable, however, is that the difference found at 16 weeks after birth was not as high as after 4 weeks after birth (four to five versus six to seven orders of magnitude) probably because of the age of mice since at 16 weeks mice seem to be less susceptible to pertussis infection (Figure 1B; cf. CFUs recovered from Cpups at 4 versus 16 weeks of age). As to the antibody titers, we performed a quantification of both the anti-whole-cell-*B. pertussis* (a-Bp) and the anti-PTxA (a-PTxA) antibodies in the mothers and in the offspring at the times indicated in Figures 1A,C lists the a-Bp- and a-PTxA-antibody titers detected in the immunized mothers and in their offspring. In contrast to the undetectable titers in the Cpups born to non-immunized females, significant levels of a-Bp and a-PTxA IgGs were present in the serum of the Ipups (Figure 1C). The antibody titers in the Ipups were lower at 16 weeks than at 4 weeks after birth, although the titers were still high enough to afford complete protection against *B. pertussis* infection (Figure 1B). With respect to the time elapsed between the administration of the last dose of vaccine to the mother and the evaluation of the protective capacity in the offspring, it is important to point out that the immunity transferred to the offspring still provided protection against *B. pertussis* infection even for the pups born to mothers whose last vaccination was given at up to 5 weeks before pregnancy (Table 1).

**Table 1:** Protection of offspring through maternal immunization before pregnancy.

	Time elapsed between the last immunization and pregnancy			
Log_10_CFU/lungs	0 weeks	1 week	2 weeks	5 weeks
Cpups	7.00 ± 0.86	7.52 ± 0.42	7.12 ± 0.54	7.72 ± 0.64
Ipups	1 ± 0.0	1 ± 0.0	1.07 ± 0.15	1 ± 0.0

*p < 0.001 Ipups versus Cpups at each tested time*.

**Figure 2:**
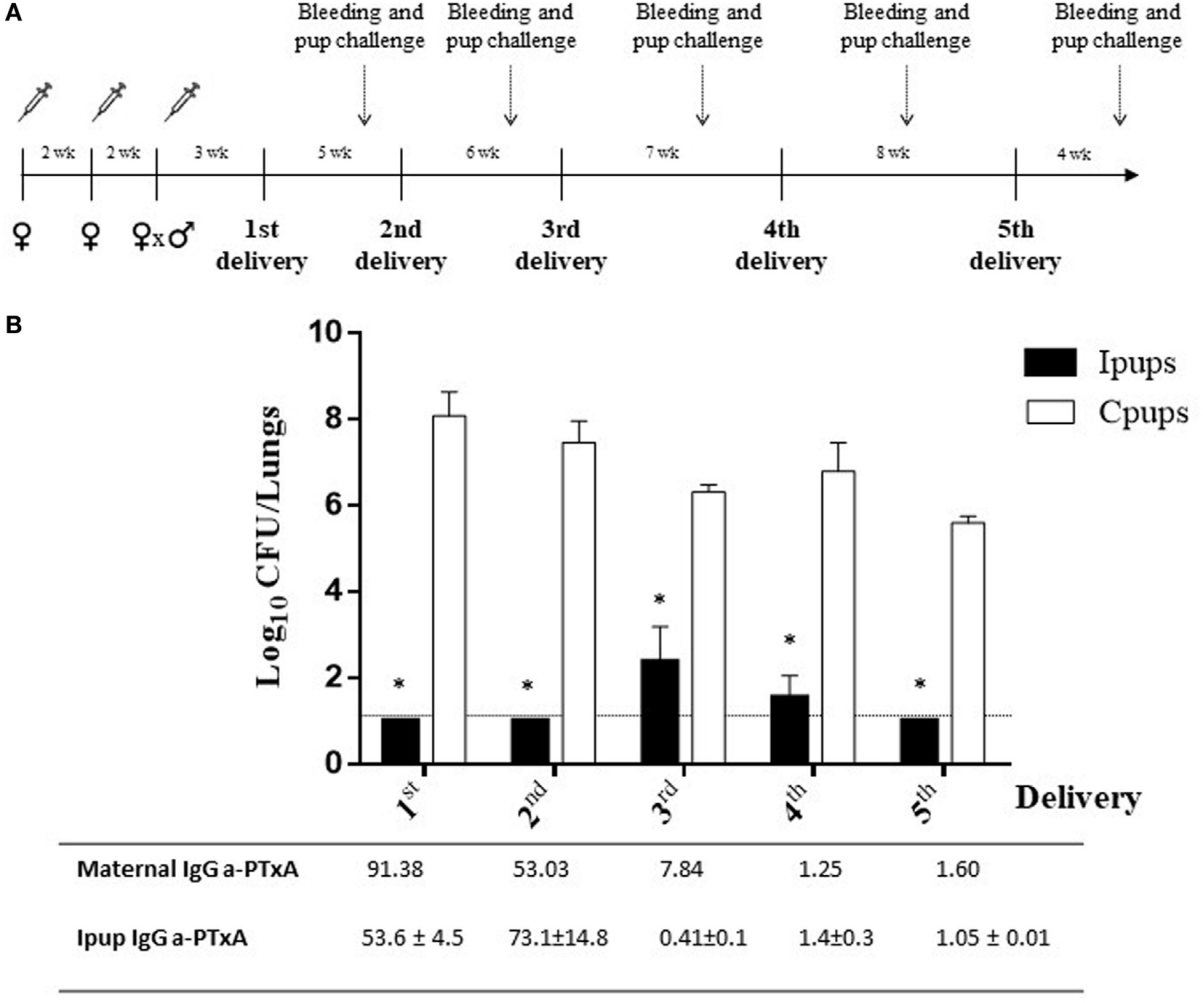
Duration of protective immunity conferred by maternal immunization beyond the first pregnancy. **(A)** Schematic representation of the maternal vaccination and pup-challenge protocol. Female mice were vaccinated twice with commercial acellular pertussis (aP) at a 2-week interval; mated with male mice; and when the vaginal plug was detected, a third aP dose was administered. Non-immunized females were mated with male mice at the same time as the immunized females. The first birth was 3–4 weeks after the last immunization. Successive births occurred at intervals of 5–8 weeks. Mice born to aP-immunized or non-immunized females (the negative controls) were challenged with *Bordetella pertussis* at 4 weeks after birth. Mothers and pups were bled 1 day before pup challenge as indicated by the dotted vertical arrows. **(B)** Protection conferred to the offspring born in later pregnancies by maternal immunity acquired during the first pregnancy. Mice born to aP-immunized (Ipups *n* = 5) or non-immunized (Cpups *n* = 5) females were challenged with *B. pertussis* Tohama I 4 weeks after birth. The protection conferred to the offspring through maternal immunization during the first pregnancy was estimated by determining the number of bacteria recovered from mouse lungs. The bacterial counts expressed as the log_10_ of CFU per lungs, is plotted on the *ordinate* as a function of the number of births after the initial delivery indicated on the *abscissa*. *p < 0.001 Ipups versus Cpups. The anti-pertussis toxin (a-PTxA) IgG titers in the mothers and the offspring determined at the time of challenge are listed below the *abscissa*. The titers are expressed as the geometric mean of the data from each group (*n* = 5). *p* < 0.001 a-PTxA IgG titers in Ipups from the first and second deliveries versus those detected in Ipups from the third through the fifth deliveries. The results from one representative experiment are presented.

Another significant result observed in relation to maternal vaccination and immune protection of the successive litters was that the immunity acquired during the first pregnancy proved to be capable of conferring protection to the offspring born in later pregnancies (Figures 2A,B). For example, we detected a protection against *B. pertussis* infection in the Ipups born in the second pregnancy in which the reduction in CFUs recovered from the lungs was by more than six orders of magnitude below the levels determined in the Cpups (Figure 2B). Moreover, we also detected differences of 3.9–5.2 orders of magnitude between the CFUs recovered in the Ipups born in the third through the fifth pregnancies and those measured in the Cpups (Figure 2B). Of particular interest to us was that the antibody titers against PTxA detected in the pups born in the later pregnancies were lower than those recovered from the pups born in the earlier ones; but nevertheless, as we observed here, those titers were still high enough to protect the pups against *B. pertussis* infection (Figure 2B). In this sense, the technique produced a degree of redundancy of protection that constitutes a benefit in this model system simulating a clinical situation in generating a certain margin of error that would be of pragmatic value in the latter circumstance.

### Donor Pups for Examination of Effector Mechanisms of Passive Immunity Transfer

**Figure 3:**
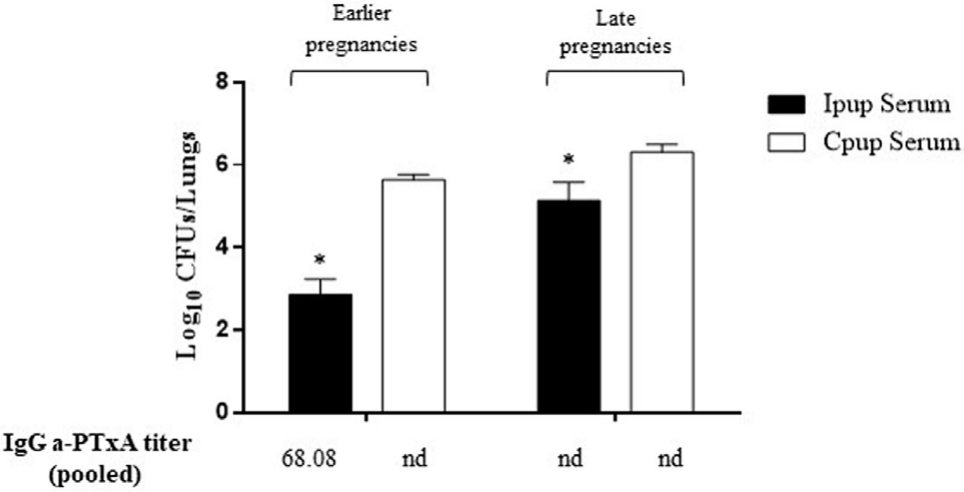
Effect of passive immunization on protection through sera collected from Ipups. Pooled sera from either early pregnancy Ipups with high anti-pertussis toxin (a-PTxA) titer or late-pregnancy Ipups with low a-PTxA titer were tested by transfer to naive (Cpup *n* = 5) female mice. Pooled sera from Cpups transferred to naive female mice were used as negative control of protection. Twenty-four hours after transfer, the mice were infected with *Bordetella pertussis* and sacrificed 7 days later to determine the CFUs in the lungs. In the figure, the number of bacteria recovered from mouse lungs, expressed as the log_10_ of CFUs per lungs, is plotted on the *ordinate* for each of the two serum-donor groups (early- and late-pregnancies) indicated above the bars. *p < 0.05 Ipup serum versus Cpup serum. The IgG a-PTxA titers are listed for each pool below the *abscissa* (nd, not detected).

To evaluate the contribution to protection of the transferred maternal antibodies, we performed transfer experiments of immune sera from Ipups to naïve mice. As a negative control of this protection, an equal volume of non-immune sera (100 µl) from Cpups was transferred to groups of five of those naïve female BALB/c mice. Twenty-four hours after transfer, all the mice were infected with a sublethal dose of *B. pertussis* and sacrificed 7 days later to determine the number of CFUs in the lungs. Transfer of 100 µl of sera from Ipups either with high titer (sera from pups from the first and second deliveries, the earlier pregnancies) or with low titer (sera from pups born from the third through the fifth deliveries, the later pregnancies) was found to confer protection (Figure 3). A reduction of 2.76 logs or 1.17 logs in CFU counts was detected relative to the control group (Figure 3) in the mice thus passively immunized with a high and a low titer of immune sera, respectively (*p* < 0.05).

**Figure 4:**
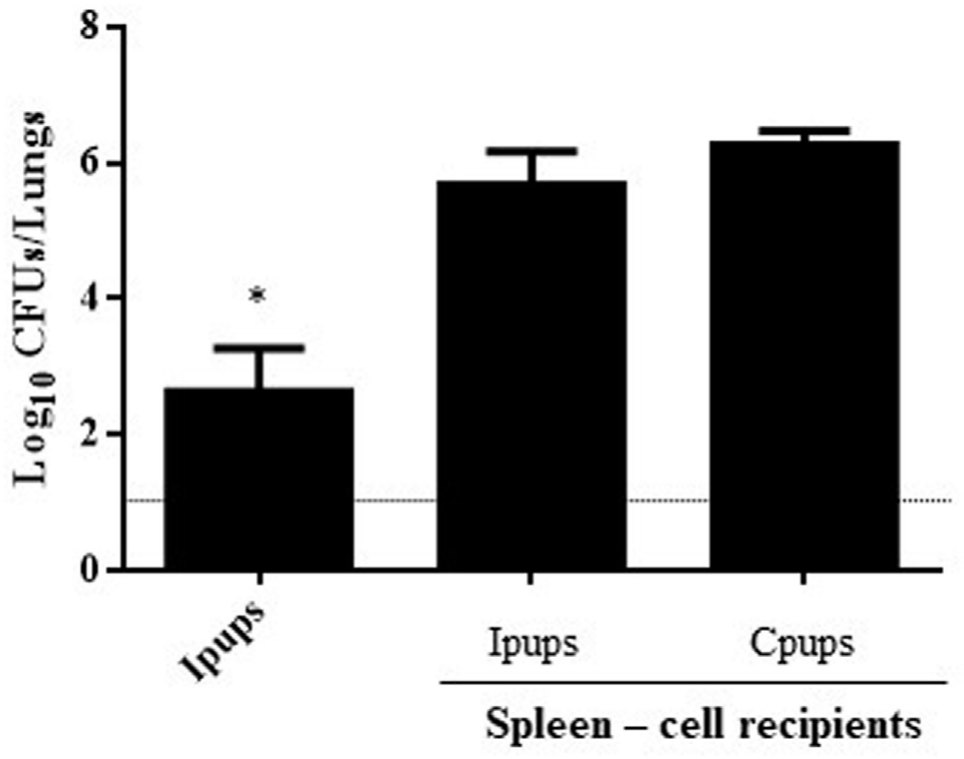
Effect of passive immunization through spleen cells collected from Ipups on protection from infection. Whole spleen cells (20–50 × 10^6^) from Ipups or Cpups (controls) were tested as possible vehicles of immune protection by passive transfer to naïve mice. Twenty-four hours after transfer, the mice were infected with *Bordetella pertussis* and sacrificed 7 days later to determine the CFUs in the lungs. Ipup-recipients were used as a positive control. In the figure, the number of bacteria recovered from the mouse lungs, expressed as the log_10_(mean CFUs ± SD of per lungs), is plotted on the *ordinate* for each of the spleen-cell-recipient groups (*n* = 7) indicated on the *abscissa*. The dotted horizontal line marks the lower limit of detection. *p < 0.05 Ipups versus both spleen-cell recipient groups.

Similar transfer experiments were also performed with spleen cells removed surgically from Ipups born from the earlier deliveries and injected into naïve mice, with the spleen cells from non-immunized mice being used as a negative control for protection. One day after the transfer, the recipient mice were challenged with *B. pertussis*, and 7 days after the challenge the CFUs in the lungs of the recipients were counted. In this instance, no significant difference was found between the control group and the group injected with spleen cells from the Ipups (Figure 4).

### Passive Immunization through Lactation

**Figure 5:**
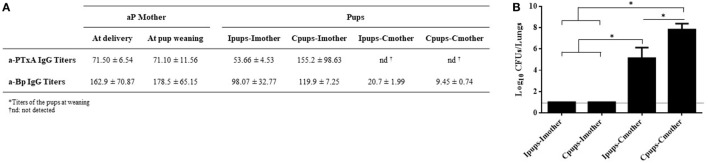
Effect of passive immunization through lactation on pertussis protection. Pups born from immune mothers (Ipups) were fostered to non-immune mothers (Ipups–Cmother), and pups born from control mothers were fostered to immune mothers (Cpups–Imother). Ipups breast-fed by their immune mother (Ipups–Imother) and Cpups breast-fed by their non-immune mother (Cpups–Cmother) were used as the respective positive and negative controls. All the pups (*n* = 6 for each group) were suckled for 21 days. **(A)** Sera from mothers and pups were collected at different time points to analyze the titers of anti-pertussis toxin (a-PTxA) and anti-whole-cell-*B. pertussis* (a-Bp) IgGs. The titers are expressed as the geometric mean of the data for each group (*n* = 6). **(B)** Pups were challenged with a sublethal dose of *Bordetella pertussis* Tohama phase I to analyze protection. In the figure, the number of bacteria recovered from the mouse lungs, expressed as the log_10_(mean CFUs ± SD of per lungs), is plotted on the *ordinate* for each of the dam-suckling groups indicated on the *abscissa*. The dotted horizontal line marks the lower limit of detection. Two independent experiments were performed. *p < 0.05 for Ipups–Imother and Cpups–Imother versus the other groups and for Ipups–Cmother versus Cpups–Cmother.

We also evaluated passive immunization through lactation. To achieve this aim, pups born to immune mothers (Ipups) were fostered to non-immune mothers (Ipups–Cmother), and pups born from control mothers were fostered to immune mothers (Cpups–Imother). Ipups breast-fed by their immune mother (Ipups–Imother) and Cpups breast-fed by their non-immune mother (Cpups–Cmother) were used as the respective positive and negative controls. After all the pups were suckled for 21 days, the pups’ sera were collected to analyze the titers of a-PTxA and a-Bp IgGs before *B*. *pertussis* challenge. Pups were then challenged with a sublethal dose of *B. pertussis* to analyze the protection against infection. Through this experimental protocol and the resulting assays, we detected that pups from the Ipups–Imother and Cpups–Imother groups exhibited high titers of a-PTxA and a-Bp IgGs (Figure 5A) and also possessed a high degree of protection against *B. pertussis*—i.e., a reduction in the CFUs recovered from the lungs by more than six orders of magnitude (Figure 5B). In contrast, pups from the group Ipups–Cmother contained low titers of a-PTxA and a-Bp IgGs and retained a low degree of protection. The lowest titers of a-PTxA and a-Bp IgGs and degrees of protection were detected in the pups from the Cpups–Cmother group (Figures 5A,B).

### Effect of Infant Vaccination in Mice Born to aP-Vaccinated Mothers on Protection

**Figure 6:**
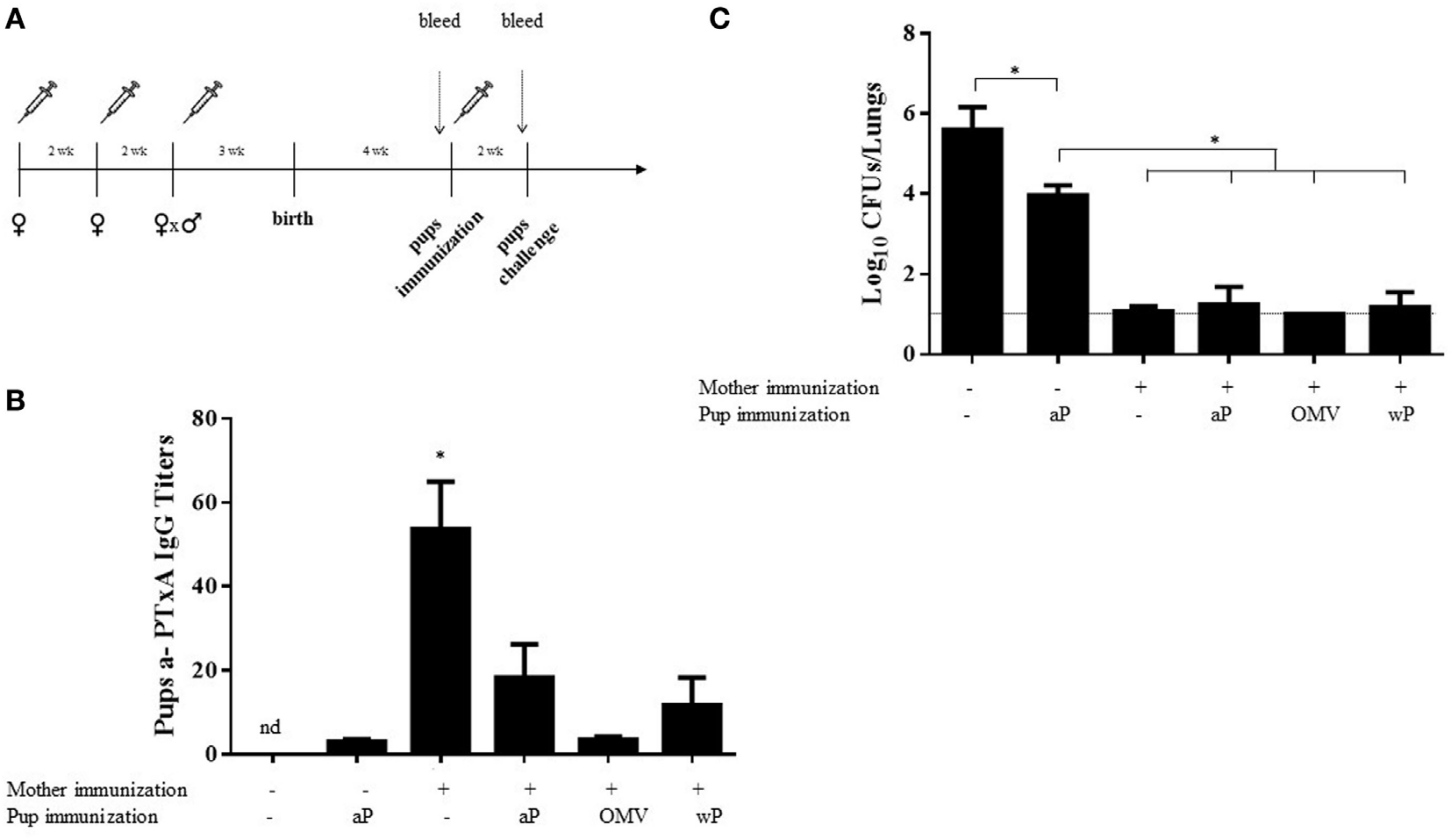
Effect of heterologous mother–pup vaccination on protection against *Bordetella pertussis* challenge. **(A)** Schematic representation of vaccination and challenge protocols. Female mice were immunized with three doses of commercial aP vaccine on days 0 and 14; mated with male mice; and when the vaginal plug was detected, a third aP dose was administered. Four weeks after birth, the offspring received either no vaccine (*n* = 8) or one dose of the aP, OMV, or wP vaccine (*n* = 8 in each group), as indicated below the *abscissas* of panels **(B,C)**. Two weeks later, the pups were challenged with *B. pertussis* and the bacterial burden in the lungs measured 7 days after challenge. Pups born to vaccinated or non-vaccinated mothers and receiving no postnatal vaccine served as controls. The pups (*n* = 5 for each group) were bled and the anti-pertussis toxin (a-PTxA) IgG titers determined 1 day before the challenge as designated by the dotted vertical arrow. **(B)** The titers, plotted on the *ordinate*, are expressed as the geometric mean of the data from each group specified on the *abscissa*. *p < 0.05 pups born to immunized mother versus others groups, and the nd signifies not detected. **(C)** In the figure, the number of bacteria recovered from the mouse lungs, expressed as the log_10_(mean CFUs ± SD per lung), is plotted on the *ordinate* for each group indicated on the *abscissa*. *p < 0.05 for negative control versus other groups, and aP-immunized pups born to non-immunized mother versus others groups. The dotted horizontal line marks the lower limit of detection. Of the three independent experiments that were performed, the results from a single representative one is presented.

To evaluate the possible interference of maternal immunization with subsequent infant immune boosting, offspring born to aP-vaccinated mothers, upon weaning from their mothers, were split into four groups at 4 weeks after birth. Three groups were treated with a single dose of either the aP vaccine, a commercial wP vaccine, or the OMV vaccine (Figure 6A), while the remaining group was left untreated. Cpups that received one dose of the aP vaccine at 4 weeks after birth were also used as control. Two weeks after vaccination of the infant mice, the antibody titers to PTxA were measured. We observed that the titers in aP-vaccinated Cpups (given a single dose) were slightly higher than those detected in the non-vaccinated Cpups. In the mice born to aP-vaccinated mothers (i.e., the vaccinated Ipups), the titers detected after vaccination with any of the three vaccines tested were lower than those quantified in Ipups left untreated (blunting effect) (Figure 6B). Moreover, that the titers in the non-vaccinated Ipups were higher than those detected in the vaccinated Cpups was most notable (Figure 6B). All the mice in this experiment, including the Cpups used as the negative control for protection, were then challenged with 10^6^–10^7^ CFUs of *B. pertussis* 2 weeks after the postnatal immunization of the Ipups. We observed that at 7 days after challenge, the vaccinated Cpups exhibited a reduced bacterial burden in the lungs by 1.5 logs (32-fold) compared with the non-vaccinated Cpups (Figure 6C). Furthermore, aP vaccination of the infant mice born to vaccinated mothers did not interfere with the maternally transmitted protective immunity, as evidenced by a comparable reduction in the CFUs detected in the lungs of approximately 5 logs compared with the burden of the non-vaccinated Cpups. In these experiments, we also observed that the immunization of Ipups with vaccines of different antigenic compositions from that used in the maternal immunization reciprocally did not interfere with the maternally transmitted protective immunity since after postnatal vaccination with either a commercial cellular vaccine (wP) or our previously designed OMV vaccine the protection conferred was similar to that seen in infant mice born to aP-vaccinated mothers with or without postnatal aP vaccination (Figure 6C).

## Discussion

The vaccination of women with aP during pregnancy is expected to provide infants with a certain degree of protection from pertussis until they are old enough to be vaccinated themselves. Because of this strategy and the data reported on its safety, the Advisory Committee on Immunization Practices (ACIP) recommended in 2011 that unvaccinated pregnant women receive a dose of tetanus toxoid, reduced diphtheria toxoid, and aP vaccine (26). In an effort to reduce the pertussis burden in infants, in 2012, the ACIP recommended the use of aP during every pregnancy (27). In their publication of August 2015, the WHO stated that they considered the vaccination of pregnant women to be most likely the greatest cost-effective additional strategy for preventing disease in infants too young to be vaccinated, with that approach appearing to be more effective and favorable than the so-called *cocooning* tactic, the vaccination of adults in close contact with infants (6). Indeed, WHO recommended that the national programs might consider the vaccination of pregnant women with one dose of aP administered during the second or third trimester or at least 15 days before delivery as a control strategy in addition to routine primary infant pertussis vaccination either in entire countries, or in other more limited settings having a high or increasing infant morbidity and/or mortality from the disease. Although the present time is still early to assess the definitive effect of implementing this strategy on the disease in infants, reports in support of that approach have already appeared in the literature (28, 29). In particular, several studies evidenced the placental transfer of anti-*B. pertussis* antibodies from aP-vaccinated mothers to their infants where the infants born to those immunized mothers had a high level of antibodies during their first months of life (28, 29). A further report in England related the key observation that vaccine effectiveness against laboratory-confirmed pertussis had been sustained during the 3 years following the vaccine’s introduction in 2012 (18). Also highly significant was the finding that the disease incidence in infants less than 3 months of age had remained low despite a high persistence in those aged 1 year and older (18). In 2017, a retrospective cohort study appeared that was designed to evaluate whether or not pertussis-infected infants born from 2011 through 2015 whose mothers had received aP vaccine during pregnancy had less severe pertussis than infants born to unvaccinated mothers. The authors concluded that the infants with pertussis whose mothers had been aP-vaccinated during pregnancy had a significantly lower risk of hospitalization and admission to intensive-care units as well as shorter hospital stays (30). Another promising aspect found in the Amirthalingam et al. (18) study was that additional protection from maternal immunization was retained in infants who subsequently received their first dose in the primary Amirthalingam series (18).

Animal models had been used earlier to obtain information about that strategy. Although the data reported once again had been scarce, evidence was nevertheless garnered for protection by the aP vaccine when used during pregnancy against intracerebral infections of *B. pertussis* and those contagions transmitted by aerosols to infant mice (22). In those studies, mice of from 6 to 10 days of age born to aP-immunized mothers were protected against an aerosol challenge with *B. pertussis* strain 18323. The authors ascertained that the protection was transferred from the dams to their offspring first through the placenta and then through the milk (22). More recently, Feunou et al. (31) confirmed that maternal immunization protected the offspring against *B. pertussis* challenge; but in their experimental paradigm, the protection waned and was eventually lost after the postnatal vaccination of the infant mice with the selfsame vaccine (31).

In the experiments reported here, we used just such a mouse model to enhance our understanding of that specific form of maternal immunization. In agreement with previous reports, we detected that maternal immunization with an aP vaccine—in our paradigm, administered in three doses (one being during the pregnancy)—led to offspring protection against *B. pertussis* infection (Figure 1). Moreover, we confirmed that the antibody levels to *B. pertussis* and PTxA were accordingly higher in those neonates (the Ipups) than in mice born to non-immunized females (the Cpups). The antibody titers in the offspring declined at 16 weeks relative to the levels at 4 weeks after birth, although the titers were high enough to protect the neonates against *B. pertussis* infection. Furthermore, the transferred antibodies, but not the spleen cells, from Ipups to naïve mice were sufficient to confer protection (Figure 3).

What was interesting to us was the observation that the immunity transferred to the offspring had a protective capacity even for pups born to mothers whose last dose of vaccine was given some weeks before pregnancy. Moreover, we detected that the immunity acquired during the first pregnancy was even capable of conferring protection to the offspring born in later pregnancies. We noted that although the titers of antibodies against PTxA were low, protection against pertussis in the Ipups born in those later deliveries was still significantly elevated (Figure 2B). These results, though having been obtained in a murine model, would underscore the need to revise the frequency with which human maternal immunization should be conducted.

In agreement with Oda et al. (22), we detected that the main protection was transferred through colostrum and/or milk. Those authors found that challenged infant mice born to mothers immunized with either the aP or the wP vaccine twice before mating evidenced the lowest increase in the number of CFUs per lung. Moreover, out of eight mice, seven deaths were registered in the non-immunized group, whereas five deaths occurred in the transplacentally immunized group, but only two in the transcolostrally immunized mice (22). In our protocol involving a schedule that included the administration of a third aP dose during pregnancy, we detected high degree of protection against *B*. pertussis through lactation (Figure 5B). In particular, we detected that the Ipups–Imother and Cpups–Imother experimental groups exhibited high a-PTxA and a-*B. pertussis* IgG titers (Figure 5A) in combination with high resulting protection against *B. pertussis* infection (more than a 6-log reduction in the CFUs recovered from the lungs: cf. Figure 5B). The results obtained by Quinello et al., in agreement with ours, demonstrated that the *pertussis*-absorbed serum and the colostrum pools protected only 30% of the immunized mice, whereas purified IgGs protected some 65% (23). Although IgA was not measured in this study or in our work, its presence could contribute at least in part to protection. In fact, it was reported that IgA induced by oral or nasally delivered pertussis antigens formulated with mucosal adjuvants confers protection although at levels not so high than the equivalent parenterally delivered vaccines [reviewed in Ref. (32)].

Although the data garnered from our murine model underscored the significance of breastfeeding in protecting infants against pertussis infection, we must point out that this protection could be less substantial in humans since in *Homo sapiens* the majority of the maternal IgG is transferred to the fetus *in utero* during pregnancy (33) and not *via* the milk.

We also observed that, when infant mice born to aP-immunized mothers (the Ipups) were vaccinated at 3 weeks of age with the same aP vaccine or a different one (i.e., a commercial wP or the OMV vaccine); the titer of IgG against a-PTx decreased (Figure 6B), but the maternally derived protection was not reduced upon that subsequent vaccination, regardless of the type of vaccine administered. These results seem to be contradictory in principle to those reported by Feunou et al., but we must bear in mind that those authors applied two vaccine boosters, one at 7 days and another at 3 weeks of life (31). The blunting effect on protection that the authors observed could have resulted from the application of those boosters—and particularly the ones performed during neonatal life—at a time when the antibody titers in the pups were still high. In contrast, our results are in agreement with those observed in humans in whom the protective capacity conferred by maternal immunization was retained in neonates receiving their first dose of the primary series (18).

Though the use of mouse models to research maternal vaccination is not expected to completely replicate human physiology, the results obtained with a model of this design will enable a test of the proposed hypotheses under controlled conditions, where the forthcoming results can then refine those hypotheses for further validation in subsequent human studies. Here, in the present mouse model, we have demonstrated that immunization with aP during pregnancy or up to 5 weeks prior effectively protects newborns against pertussis. Although the titer of maternal antibodies in the infant offspring diminishes with time, protection is not reduced for at least up until 4 months of age. Moreover, subsequent vaccination of the infant mice with the same vaccine or a different one from the type used during pregnancy did not affect the transferred maternal protection (Figure 6). The potential blunting of protection conferred by maternal immunization through infant vaccination could be developed depending on the antibody levels in the infants. Another important finding here described was that maternal-vaccination-acquired immunity from the first pregnancy still conferred protection to offspring up to the fourth pregnancy. The results presented here reinforce the need to continue studying that blunting effect in humans as well as to revise the frequency of vaccination in successive pregnancies according to the time between each one.

## Ethics Statement

The animal experiments were authorized by the Ethical Committee for Animal Experiments of the Faculty of Science at La Plata National University (approval number 004-06-15 and 003-06-15).

## Author Contributions

DH planned the study, made the laboratory analysis, interpreted data, and drafted manuscript. DB, MG, and MZ planned the study, interpreted data, and revised figures and the manuscript. DS-M, EB, FC, PA, CC, and MSB performed certain experiments and laboratory analyses. All authors approved the final manuscript.

## Conflict of Interest Statement

The authors declare that the research was conducted in the absence of any commercial or financial relationships that could be construed as a potential conflict of interest.
